# Performance of Deaf Participants in an Abstract Visual Grammar Learning Task at Multiple Formal Levels: Evaluating the Auditory Scaffolding Hypothesis

**DOI:** 10.1111/cogs.13114

**Published:** 2022-02-21

**Authors:** Beatrice Giustolisi, Jordan S Martin, Gesche Westphal‐Fitch, W. Tecumseh Fitch, Carlo Cecchetto

**Affiliations:** ^1^ Department of Psychology University of Milan‐Bicocca; ^2^ Institute of Evolutionary Medicine University of Zurich; ^3^ Department of Behavioral and Cognitive Biology University of Vienna; ^4^ Structures Formelles du Langage (Unité Mixte de Recherche CNRS and Université Paris 8)

**Keywords:** Visual artificial grammar learning, Mildly context‐sensitive grammars, Sequencing, Deafness, Auditory scaffolding hypothesis

## Abstract

Previous research has hypothesized that human sequential processing may be dependent upon hearing experience (the “auditory scaffolding hypothesis”), predicting that sequential rule learning abilities should be hindered by congenital deafness. To test this hypothesis, we compared deaf signer and hearing individuals’ ability to acquire rules of different computational complexity in a visual artificial grammar learning task using sequential stimuli. As a group, deaf participants succeeded at all levels of the task; Bayesian analysis indicates that they successfully acquired each of several target grammars at ascending levels of the formal language hierarchy. Overall, these results do not support the auditory scaffolding hypothesis. However, age‐ and education‐matched hearing participants did outperform deaf participants in two out of three tested grammars. We suggest that this difference may be related to verbal recoding strategies in the two groups. Any verbal recoding strategies used by the deaf signers would be less effective because they would have to use the same visual channel required for the experimental task.

## 1 Introduction

Humans excel at learning abstract patterns without explicit teaching, often with only minutes of exposure to a set of sequences that follow a target pattern or rule. This holds true from infancy to adulthood, and for a wide variety of different stimuli across multiple modalities (e.g., Gomez, 2002; Marcus et al., 1999; Saffran et al., 1996, 2007).

However, based on deficits in sequential processing seen in some deaf participants, Conway and colleagues proposed an influential hypothesis (Conway et al., 2009), the “auditory scaffolding hypothesis,” which states that the development of general cognitive abilities related to representing temporal and sequential patterns is directly sustained by hearing experience. The theoretical basis for this intriguing idea is that temporal and sequential changes are the foundations of sound stimuli and thus play a more crucial role in auditory than visual cognition. The socioclinical and political implications of this hypothesis are not trivial, because sequencing and timing behavior are basic building blocks for many higher cognitive functions (Lashley, 1951). If the auditory scaffolding hypothesis is indeed correct, swift action should be taken to ensure each deaf newborn has his/her sense of hearing technologically restored as soon as possible after birth, independently of the language policy adopted.

As evidence supporting the auditory scaffolding hypothesis, a group of deaf children with cochlear implants showed no learning effect in an implicit sequence learning task (Conway et al., 2011). Specifically, this group of deaf children with cochlear implants, all born in hearing families, and a control group of hearing children matched in age took part in an implicit sequence learning test with visual stimuli. Participants were asked to memorize and reproduce sequences of colored squares presented one by one on a computer screen. Without informing the participants, the task was divided into two phases, with an initial exposure phase followed by a testing phase. During the exposure phase, color sequences followed specific constraints, that is, transition probabilities between colors were fixed. During the testing phase, half of the sequences followed the constraints of the exposure phase (familiar stimuli), and half of the sequences did not (unfamiliar stimuli). Implicit sequence learning was assessed by comparing accuracy in reproducing familiar stimuli with accuracy in reproducing unfamiliar stimuli, with the difference in accuracy between familiar and unfamiliar patterns used to indicate whether implicit learning occurred. While the control hearing group showed a learning effect, the deaf children did not, providing support for the auditory scaffolding hypothesis.

However, the validity of the auditory scaffolding hypothesis has been called into question by several recent studies investigating sequence learning abilities in deaf children and adults (Giustolisi & Emmorey, 2018; Hall et al., 2018; von Koss Torkildsen et al., 2018 and Terhune‐Cotter et al., 2021). From a theoretical perspective, Hall et al. (2018) contested the validity of drawing inferences on the effects of auditory deprivation from a population of deaf children of hearing parents, who were exposed to sound via cochlear implants. In fact, in this population, the period of auditory deprivation mostly overlaps in time with a period of language deprivation. To tease apart the influence of language deprivation versus auditory deprivation on sequencing skill development, Hall et al. evaluated the auditory scaffolding hypothesis in a third group of participants: deaf children without any delay in language exposure, that is, deaf children of deaf parents, exposed to natural language (a sign language) from birth. Moreover, Hall and colleagues raised some concerns about the experimental paradigm used in Conway, et al. (2011). As previously mentioned, Conway and colleagues’ testing phase consisted of showing the children a series of sequences one at time and asking them to reproduce each sequence. The underlying hypothesis was that children should reproduce the sequences of the same type as those presented during familiarization better than unfamiliar sequences. But Hall and colleagues point out that children with high working memory span should show no learning effects, simply because their performance would be at ceiling for both types of sequences (i.e., they can correctly remember familiar sequences as well as nonfamiliar ones), meaning that learning effects would be detectable only in those children who failed to remember the unfamiliar presented sequences.

Hall and colleagues’ empirical results directly challenged the auditory scaffolding hypothesis. First, they failed to replicate the results of Conway et al. (2011): neither hearing children, deaf signing children of deaf parents, nor deaf children of hearing parents, showed significant evidence of learning using Conway's implicit sequential learning task. However, using a serial reaction time task (Nissen & Bullemer, 1987), all the three groups of children showed learning effects. In the serial reaction time task, participants had to provide different responses depending on the position of a target item. Unbeknown to participants, item position is determined by fixed transitional probabilities between possible locations. Learning these fixed transitional probabilities leads to reduced reaction times. Interestingly, the fact that deaf children of hearing parents showed learning effects also argues against a possible *language* scaffolding hypothesis, that is, that the development of implicit learning skills may depend on the temporal, hierarchical, and inherently social structure of language (Hall et al., 2018).

Also Terhune‐Cotter et al. (2021) assessed the validity of the auditory scaffolding hypothesis with a sequence learning task roughly similar to that used in Conway et al. (2011). Participants were asked to repeat sequences of either colored (easily nameable) or monochromatic (less easily nameable) shapes placed in four possible screen locations. Sequences might build upon the preceding one (repeating sequences) or not (random sequences). The comparison between repeating and random sequences was used as an index of sequence learning. In this case, participants were a group of deaf signing children and a control group of hearing non‐signing children. Both groups showed sequence learning, and no significant difference was found between the deaf children and the control group.

Von Koss Torkildsen et al. (2018) assessed implicit learning of embedded triplets of unfamiliar alien figures (see Arciuli & Simpson, 2011, 2012) in a group of prelingually deaf children with cochlear implants and a control group of hearing peers. The exposure phase was composed of a continuous stream of stimuli with embedded triplets, with participants engaged in a cover task (i.e., press a button upon the repetition of the same alien twice). In the testing phase, participants had to choose between two triplets, one that was familiar (i.e., already presented in the exposure phase) and one that was novel (i.e., never presented in the exposure phase but composed of the same aliens). Results showed that the performance of deaf children with cochlear implants was comparable to that of hearing children. In addition, in the deaf group the correlation between sequence learning performance and age of implantation or speech perception level was not significant. The author's discussion focused on the difference between the stimuli used in their task (pictures of aliens) versus those used in Conway et al. (2011) (colored squares). von Koss Torkildsen et al. (2018) suspect that differences in verbal rehearsal strategies between deaf children with cochlear implant and hearing children might play a key role in determining the results of Conway et al. (2011) (it is likely that participants verbalized colored squares, whereas it is unlikely that they could verbalize unfamiliar alien figures).

The proposition that the human ability to learn abstract rules in the visual modality without explicit teaching is independent from hearing experience is also supported by results from Giustolisi and Emmorey (2018). To assess sequential learning in deaf adults with a lifelong lack of hearing experience but early exposure to a sign language, they used a version of the triplet paradigm (see Siegelman et al., 2017) in which the stimuli were composed of abstract black shapes. Contrary to the auditory scaffolding hypothesis, the vast majority of participants performed above chance level, showing a clear learning ability for sequence regularities without explicit teaching.

**Fig. 1:**
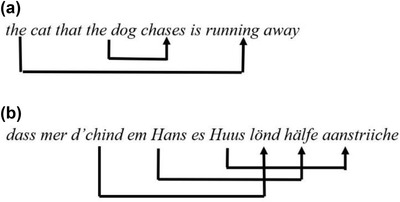
(a) Example of nested dependencies in natural language syntax. (b) Example of crossed dependencies in natural language syntax. The sentence, taken from Shieber (1985), is in Swiss German and its meaning is “that we let the children help Hans paint the house”.

The studies described so far assessed implicit learning of simple patterns involving fixed transition probabilities across stimuli. They have investigated the ability of grouping together continuous items, which is one specific level of abstraction through which sequences can be coded, but not the only one. Following the taxonomy of sequence knowledge proposed by Dehaene et al. (2015), at least four additional different systems are needed to represent sequencing abilities, each system based on a different degree of abstraction. Considering this multifaced nature of sequence knowledge, and the beforementioned socioclinical implications of the auditory scaffolding hypothesis, in our opinion an evaluation at different levels of sequence processing is crucial. The goal of the present work was to focus on tree structures with nested and crossed dependencies, which, compared to previous works, is a more abstract level through which sequences can be coded. This level of abstraction is required to account for the processes that we find in one of the more complex types of sequence that we use every day, that is language. Our aim was to evaluate whether a lack of hearing experience really hinders learning of sequential patterns at a higher level of complexity, as expected following the auditory scaffolding hypothesis. We know from previous research that human implicit learning abilities are not restricted to simple regularities but extend to more complex patterns generated by different types of artificial grammars (Fitch & Hauser, 2004; Uddén et al., 2012; Westphal‐Fitch et al., 2018). Using formal language theory, grammars can be ranked at different level of complexity on the so‐called extended Chomsky hierarchy (Jäger & Rogers, 2012). The simplest level in the hierarchy is that of regular grammars (e.g., sequential transition probabilities), with the next level of complexity given by context‐free grammars, which also allow the processing of nested dependencies. Natural language syntax requires computations at a still higher level of the extended hierarchy, that of mildly context‐sensitive grammars, which allow the processing of both nested and crossed dependencies. Center embedded relative clauses (Fig. 1a) are an example of nested dependencies, whereas crossed dependencies (Fig. 1b) can be found in languages like Swiss German (the example in Fig. 1b is from Shieber, 1985). Context‐free and context‐sensitive grammars are collectively termed “supra‐regular” grammars.

The current study builds on previous work by Westphal‐Fitch et al. (2018), who used a classic artificial grammar learning paradigm to assess visual pattern‐processing skills in a group of 20 university students. Participants were exposed for several minutes to visual sequences made up of abstract tile patterns, generated by grammars at different formal levels. Participants were instructed simply to observe the sequences, without being involved in any cover task. Then, in a successive testing phase, they were asked to judge if novel strings were similar to that seen in the exposure phase or not. The artificial grammars that were used spanned all levels relevant for human language, that is, included a regular grammar (AB^N^A), a context‐free grammar (Mirror grammar), and a mildly context‐sensitive grammar (Copy grammar). The AB^N^A grammar generates strings beginning and ending with an A element, with a variable number of B elements in the middle (e.g., A BB A; A BBBBB A), with a simple long‐distance dependency between the two As. The Mirror grammar generates strings in which the second half‐sequence is a reversal of the first (e.g., AAB BAA; ABAA AABA), leading to nested dependencies between its elements. The Copy grammar generates strings in which the second half simply reduplicates the first (e.g., AAB AAB; ABAA ABAA), creating crossed dependencies.

Participants were able to learn all three grammars: they correctly categorized as “similar” strings following the same pattern as exposure stimuli (including extensions, i.e., strings of a length not used in the exposure phase, thus showing generalization), and as “dissimilar” ungrammatical foils with incomplete dependencies. Successful learning was observed both at the group level, and individually for almost all participants.

In this study, we tested the auditory scaffolding hypothesis using the paradigm of Westphal‐Fitch et al. (2018). Specifically, we assessed the ability of deaf adults to acquire artificial grammars presented visually that differed in complexity. We investigated participants’ ability both to recognize familiar stimuli, and to generalize the acquired rule to strings of different lengths than the lengths presented in the exposure phase. Moreover, we tested a control group of hearing participants. Our primary aim was to determine if hearing and deaf participants differ in the strategies they use to perform the task, but also to provide a possibility to replicate Westphal‐Fitch et al.’s (2018) findings in a different group of participants.

## 2 Method

### 2.1 Participants

One group of deaf people and one group of hearing people participated. Deaf participants were 15 Italian Sign Language (LIS) signers (*M*_age_ = 33 years; *SD* = 14 years; range = 18–62; seven females, eight males) recruited from the members of four Italian Deaf Institutes located in Milan, Monza, Turin, and Verona. They were all born deaf and none of them had any associated disability or further sensory deficits. Seven out of 15 deaf participants (47%) were native signers, exposed to LIS from birth, while eight out of 15 (53%) were first exposed to LIS during childhood or adolescence. At testing, all participants were fluent LIS signers and used LIS as their main everyday means of communication. They also used Italian with different degrees of proficiency. The mean number of years of education was 13.7 years (*SD* = 2.6). One additional deaf participant was excluded as they scored below the normal range in the Raven's test following Basso et al. (1987). Hearing participants were 15 hearing Italian speakers (seven females, eight males) with no knowledge of LIS or any other sign language, recruited through the Sona System platform of the Milan‐Bicocca University or through online social media. They were matched with the deaf participants in age (*M*_age_ = 34 years; *SD* = 15 years; range = 18–59; *t*(28) = −0.16, *p* = .87) and level of education (*M*_years_edu_ = 13.5 years; *SD* = 2.4; *t*(28) = −0.07, *p* = .95). Overall cognitive abilities of the two groups of participants were assessed using Raven's Colored Progressive Matrices (Raven, 1965). Raw scores were corrected following Basso et al. (1987). Corrected scores mean for the deaf participants was 31.67 (*SD* = 4.76), for the hearing participants was 33.33 (*SD* = 3.11), with no significant difference between groups (*t*(28) = −1.14, *p* = .27, *d* = −0.42 95% CI [−1.14, 0.31]). Visuospatial working memory span was assessed with the Corsi‐block tapping task (Corsi & Michael, 1972). The task was administered using the nine square blocks positioned on a plastic board. The mean span for the deaf participants was 5.67 (*SD* = 0.90) and for the hearing participants 5.53 (*SD* = 0.99); this difference was not significant (*t*(28) = 0.38, *p* = .70, *d* = 0.14 95% CI [−0.58, 0.86]). All participants had normal or corrected‐to‐normal vision. No participant had previous experience with experimental psychology investigations. Participants gave their written informed consent prior to taking part to the experiment and received 20€ reimbursement for their participation. The study was approved by the ethics committee of the University of Milan‐Bicocca and was carried out in accordance with the code of ethics of the World Medical Association (Declaration of Helsinki).

### 2.2 Materials and procedure

The visual grammar learning approach built on previous work, using short strings of complex‐colored tiles to probe pattern perception abilities. Previous research using such stimuli included both humans and animals (Stobbe et al., 2012), and the current study was closely based on an earlier publication using the same stimuli with hearing participants (Westphal‐Fitch et al., 2018). Because this approach uses purely visual stimuli, but otherwise matches considerable previous work using auditory or written stimuli (Fitch & Friedrici, 2012; Reber,1967; Saffran, 2002), it offers an ideal test bed for investigating pattern learning abilities in deal participants.

Please refer to Westphal‐Fitch et al. (2018) for a detailed explanation of materials and procedure. Here, a brief summary is provided. The experimental stimuli are available through the OSF repository of the present project (https://osf.io/25det/).

#### 2.2.1 Grammars and stimuli

The “warm‐up” grammar was a regular grammar (AB)^N^ that generated sequences of (AB) elements. The testing grammars were three different grammars with long distance dependencies, located on different levels of the extended Chomsky hierarchy: a regular grammar (AB^N^A), a context sensitive grammar (Mirror grammar), and a mildly context‐sensitive grammar (Copy grammar). The sequences generated by the grammars were composed of colorful abstract decorated small squares (*tiles*) sized 20 × 20 pixels, clearly belonging to two different categories. Sequence's structure was marked by black rectangles (black tiles) of 16 × 20 pixels (two for AB^N^A sequences, between A and B elements, and one for Mirror and Copy sequences, in the middle). Each tile was sequentially presented against a black background one at a time, one new tile every 166 ms. New tiles were presented adjacent to the location of the previous tile, and tiles remained on the screen until the entire sequence was completed. Thus, the sequences appeared on the screen as if typed on a typewriter. Then the whole sequence disappeared, and the screen remained blank until the participant responded.

#### 2.2.2 Procedure

**Fig. 2:**
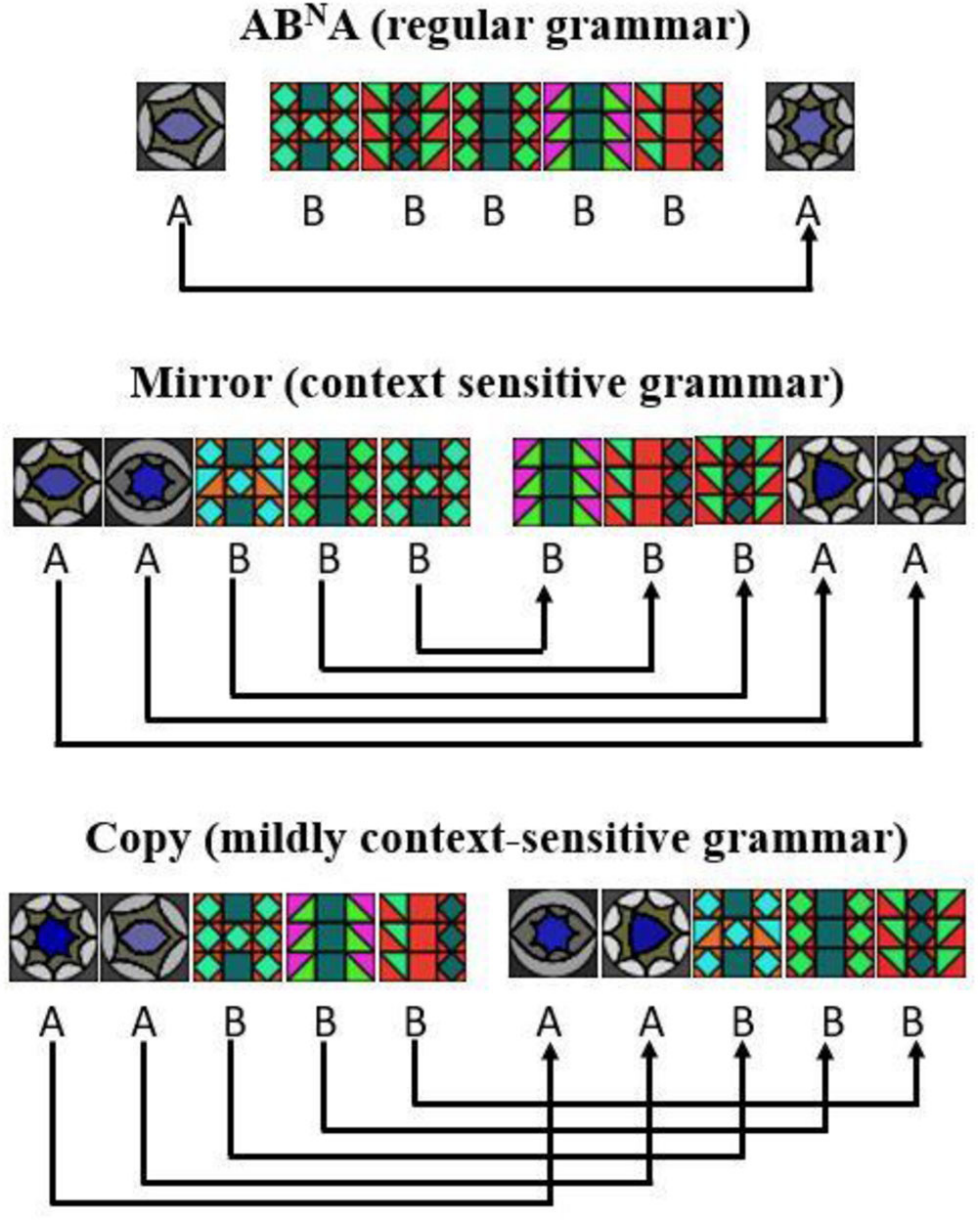
Examples of grammatical sequences for all three grammars with *N* = 5.

All participants received instructions in written Italian, deaf participants were additionally provided with instructions in LIS (video instructions) so that all participants could receive instruction in their preferred language. After completing the warm‐up task (using the (AB)^N^ grammar), each participant was tested on the three target grammars in a randomized order. For each grammar, the procedure was divided in two phases: exposure and subsequent testing. During each phase, all participants saw the same sequences, but in a different randomized order. During exposure (duration approx. 2 min), participants saw 30 grammatical sequences with *N* = 2, 3, and 5^1^ (Fig. 2 shows example of exposure sequences with *N* = 5). During testing, participants saw 87 individual strings: 36 grammatical (*N* = 2 and *N* = 3, and extensions to *N* = 4 and *N* = 6) and 51 ungrammatical (*N* = 2, 3, 4 and 6). Ungrammatical “foil” strings included sequences with a missing element, and sequences with the correct number of elements, but incorrect category membership (see Westphal‐Fitch et al. (2018), table 1, for more detailed information about the stimuli). The participants’ task in the test phase was to indicate whether the sequence followed the same schema as those seen during the exposure phase or not by pressing a yes/no key on a keyboard. Response time was not limited and no feedback was given. Participants were not explicitly asked to report the rule(s) they used to perform the task and no participant (neither deaf nor hearing) spontaneously did it. To ensure that participants understood the task and were paying attention, the experimental session was preceded by a training session during which participants were exposed to the warm‐up regular grammar, (AB)^N^. Success on this grammar (accuracy > chance level, i.e., accuracy > 12/15, Exact binomial test, *p* = .02) was a prerequisite for taking part in the experimental session and it was achieved by all participants.

**Table 1:** Individual participant performance on novel grammatical stimuli and ungrammatical foils with missing dependencies. Raw number and percentage of participants who reach the criterion value in the three grammars shown separately for the *N* conditions

	Deaf participants (total = 15)			Hearing participants (total = 15)		
Condition	AB^N^A	Mirror	Copy	AB^N^A	Mirror	Copy
*N* = 2 and 3	14 (93%)	14 (93%)	14 (93%)	15 (100%)	15 (100%)	14 (93 %)
*N* = 4	7 (47%)	8 (53%)	9 (60%)	14 (93%)	10 (67%)	10 (67%)
*N* = 6	11 (73%)	1 (7%)	0 (0%)	15 (100%)	3 (20%)	4 (27%)

All participants were tested using the same laptop (Fujitsu LIFEBOOK A Series, 15.6 inches screen, 4th generation Intel Core i7 processor) running Windows 8, whereas the testing rooms differed based on the location of the participant. In all cases, testing rooms were quiet, and occupied only by the participant and the experimenter.

## 3 Results and analysis

We first considered the acquisition of the grammars by analyzing the percentage of “yes” responses (i.e., same schema) for grammatical and ungrammatical stimuli at the group level and individually. Responses to grammatical and ungrammatical stimuli were compared using Wilcoxon signed‐rank test. We analyzed the performance of the two groups separately, and of participants individually. Then, accuracy data were analyzed using a multilevel Bayesian modeling framework developed in Westphal‐Fitch et al. (2018). We assessed whether participants performed the task by effectively using the target grammar. Finally, we directly compared the performances of deaf and hearing participants. Data and code to perform the Bayesian multilevel analysis are available in the second author's GitHub repository (https://github.com/Jordan‐Scott‐Martin/AGL‐studies).

### 3.1 Overall group performance

The first analysis dealt with the participants’ ability to accept grammatical strings and reject ungrammatical foils. Deaf participants correctly responded “same” to 81% (*SD* = 19) of AB^N^A grammatical strings, and incorrectly responded “same” 17% (*SD* = 19) of AB^N^A ungrammatical foil strings (*W* = 120, *p* < .001, Cohen's *d* = 2.52, *d′* = 1.85). Considering the Mirror grammar, they responded “same” to 82% (*SD* = 18) of grammatical and 35% (*SD* = 22) of ungrammatical strings (*W* = 120, *p* < .001, Cohen's *d* = 1.73, *d′* = 1.31). As for the Copy grammar, “same” responses were given to 82% (*SD* = 18) of grammatical and 34% (*SD* = 20) of ungrammatical strings (*W* = 120, *p* < .001, Cohen's *d* = 1.80, *d′* = 1.32).

Turning to hearing participants, in the AB^N^A grammar they responded “same” to 94% (*SD* = 9) of grammatical and 5% (*SD* = 7) of ungrammatical strings (*W* = 120, *p* < .001, Cohen's *d* = 6.10, *d′* = 3.22). In the Mirror grammar, “same” responses were given to 84% (*SD* = 15) of grammatical and 21% (*SD* = 19) of ungrammatical strings (*W* = 120, *p* < .001, Cohen's *d* = 2.05, *d′* = 1.76). As for the Copy grammar, hearing participants responded “same” to 82% (*SD* = 18) of grammatical and 18% (*SD* = 19) of ungrammatical strings (*W* = 120, *p* < .001, Cohen's *d* = 2.15, *d′* = 1.81). To summarize, both groups succeeded at learning each of the three grammars: both the deaf and the hearing group correctly accepted novel grammatical sequences and rejected ungrammatical foils.

**Fig. 3:**
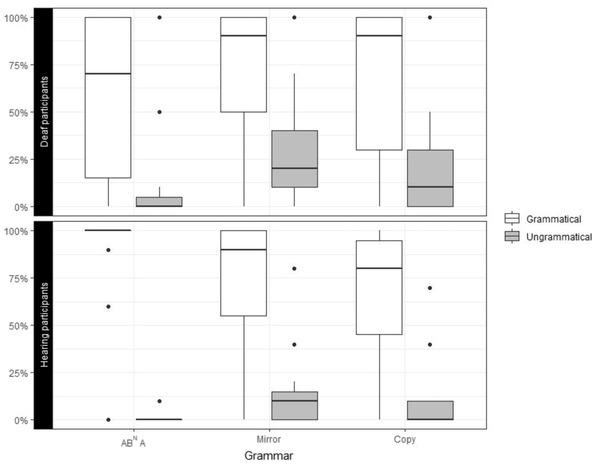
Percentage of “yes” responses (= same schema) for novel grammatical *N* = 4 sequences (white) and ungrammatical sequences with incomplete dependencies (gray) in the three target grammars. Upper panel: deaf participants, lower panel: hearing participants.

We then focused on participants’ ability to generalize to stimuli of novel lengths. In the exposure phase, participants saw sequences of *N* = 2, 3, or 5. In the testing phase, we included sequences of *N* = 4 (generalization to an intermediate length) and sequences of *N* = 6 (generalization to longer strings). Both groups showed the ability to generalize to an intermediate length in all three grammars (Fig. 3. Deaf participants–AB^N^A: *W* = 76.5, *p* = .003, Cohen's *d* = 1.03, *d′* = 1.43; Mirror grammar: *W* = 65, *p* = .005, Cohen's *d* = 0.97, *d′* = 1.12; Copy grammar: W = 63, *p* = .008, Cohen's *d* = 1.04, *d′* = 1.18. Hearing participants – AB^N^A: *W* = 105, *p* < .001, Cohen's *d* = 3.29, *d′* = 3.40; Mirror grammar: *W* = 78, *p* = .002, Cohen's *d* = 1.51, *d′* = 1.70; Copy grammar: *W* = 78, *p* = .002, Cohen's *d* = 1.47, *d′* = 1.79).

**Fig. 4:**
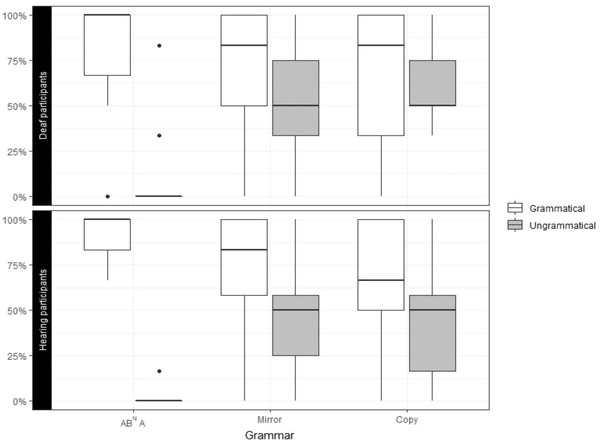
Percentage of “yes” responses (= same schema) for novel grammatical *N* = 6 sequences (white) and ungrammatical sequences with incomplete dependencies (gray) in the three target grammars. Upper panel: deaf participants, lower panel: hearing participants.

Both deaf and hearing participants showed the ability to generalize to longer strings of *N* = 6 in the regular AB^N^A grammar. However, only the hearing participants showed *N* = 6 generalization in the two supra‐regular grammars (Fig. 4). Deaf participants – AB^N^A: *W* = 105, *p* < .001, Cohen's *d* = 2.17, *d′* = 2.26; Mirror grammar: *W* = 70.5, *p* = .09, Cohen's *d* = 0.55, *d′* = 0.37; Copy grammar: *W* = 35.5, *p* = .86, Cohen's *d* = 0.17, *d′* = 0.11. Hearing participants – AB^N^A: *W* = 120, *p* < .001, Cohen's *d* = 7.35, *d′* = 3.52; Mirror grammar: W = 71.5, *p* = .012, Cohen's *d* = 0.81, *d′* = 0.78; Copy grammar: *W* = 89.5, *p* = .022, Cohen's *d* = 0.73, *d′* = 0.71).

### 3.2 Individual above‐chance performance

The performance of participants of the two groups was also analyzed at an individual level, considering success in each grammar separately for varying *N* (see Table 1). Success was determined by comparing the number of correct answers by participants with a criterion calculated using a binomial test. For new grammatical sequences and ungrammatical foils with incomplete dependencies, “success” corresponded to at least 20/30 trials correct (exact binomial test, *p* = .049). For grammatical and ungrammatical sequences with incomplete dependencies of *N* = 4 the criterion was at least 15/20 correct (*p* = .02) and considering *N* = 6 at least 10/12 correct (*p* = .02).

As Table 1 shows, almost all participants’ performance was above chance level with novel strings of *N* = 2 and *N* = 3. With *N* = 4 strings, performance above chance level was observed in about half of the deaf participants, in 10/15 hearing participants in the two supra‐regular grammars, and almost all hearing participants (14/15) in the regular grammar. All hearing participants and the majority of deaf participants (11/15) performed above chance level with *N* = 6 stimuli in the regular grammar, whereas performance with *N* = 6 stimuli in the supra‐regular grammars was poor for both groups, with almost no deaf participant performing above chance level, and only three to four hearing participants doing so.

We also considered individual performance on ungrammatical stimuli with a single incorrect tile. “Success” for these ungrammatical sequences of *N* = 2 or 3 was counted as at least 12/15 correct “different” answers (*p* = .02), and for sequences of *N* = 4 as at least 9/10 correct answers (*p* = 0.01). Results are reported in Table 2. Most of the deaf and hearing participants performed above chance level in the regular grammar, whereas 40%–50 % of deaf participants and 53%–73% of hearing participants performed well in the two supra‐regular grammars,

**Table 2:** Individual participant performance on ungrammatical stimuli with an incorrect tile. Raw number and percentage of participants who reached the criterion value in the three grammars separately for each *N* condition

	Deaf participants (total = 15)			Hearing participants (total = 15)		
Condition	AB^N^A	Mirror	Copy	AB^N^A	Mirror	Copy
*N* = 2 and 3	10 (67%)	6 (40%)	6 (40%)	12 (80%)	9 (60%)	11 (73%)
*N* = 4	10 (67%)	7 (47%)	7 (47%)	14 (93%)	8 (53%)	11 (73%)

### 3.3 Bayesian multilevel analysis

Our previous analyses show that both hearing and deaf participants perform above‐chance in most conditions, indicating that participants were sensitive to some structural properties of the grammatical stimuli. However, these results do not necessarily demonstrate induction of the intended AB^N^A, Mirror, or Copy target grammars, as individuals can potentially utilize a variety of alternate rules, including “short cuts” at different levels of grammatical complexity (Fitch & Friederici 2012, O'Donnell et al., 2005). If such alternative strategies exhibit correlated dependencies with the target grammar across specific subsets of experimental stimuli, statistically significant performance can be attained with an alternate rule. For example, a participant simply selecting all stimuli ending in A elements (a regular rule) would perform similarly to a participant using the supra‐regular Mirror grammar for those stimuli that satisfy both rules (e.g., ABBA, ABBBBA, ABABBABA). To address this issue, we used a Bayesian multilevel model developed by Westphal‐Fitch et al. (2018) to estimate the probability of each participants’ performance being consistent with the induction of the target grammars versus a plausible set of alternate grammars. This was accomplished by coding each trial's response as being consistent (1) or inconsistent (0) with each possible grammar, and then comparing the estimated probabilities or odds of grammar‐consistent responses across the entire set of responses and stimuli. The alternative grammars are listed in the supplementary materials. We implemented these multilevel models within a Bayesian framework using Markov Chain Monte Carlo (MCMC) estimation, allowing hypothesis testing at both the individual and group level, based upon posterior probability distributions.

Here we applied this framework to determine (i) whether deaf and hearing participants’ responses were more consistent with induction of the target grammars as compared to the alternate grammars, as well (ii) whether deaf and hearing participants differed in their overall performance and responses toward key sequence properties. Using Bayesian logistic regression models with random subject‐level effects, we first estimated and compared the probability of the average deaf and hearing participant performing consistently with each of the target and alternate grammars. While absolute probabilities greater than chance (*p* > .50) indicate that performance is nonrandomly associated with application of the test grammar, higher probabilities indicate a great chance that participants were in fact applying a specific grammar rule consistently across trials (e.g., *p* = .90 suggests that responses are on average consistent with a grammar in 90% trials, while *p* = .6 indicates nonrandom performance but consistent application of the rule in only 60% trials). Given that true and alternate grammars are both satisfied for particular subsets of experimental stimuli, and some participants may not apply the same rules consistently across all trials, it is expected that some alternate grammars may have above‐chance probabilities of consistent responses. To address this issue, we further assessed whether deaf and hearing participants performed more consistently with the target grammar as compared to alternate grammars. Log odds ratios (LogOR) were used for these comparisons, so that LogOR = 0 indicated equivalent odds of performing consistent with a target and alternate grammar, while LogOR > 0 indicated greater support for the target grammar. Individual random intercepts were further used to assess patterns of individual variation unexplained by the average differences among hearing and deaf participants.

All analyses were done using the brms package (Bürkner, 2018), which facilitates Bayesian modeling using the Stan statistical programming language (Carpenter et al., 2017) in the R statistical environment (R Core Team, 2013). Weakly regularizing priors—Normal(0,2) for fixed effects, Half−Cauchy(0,2) for random effects, and LKJ(2) for correlation coefficients—were set on all model parameters to reduce the risk of false positives and facilitate more robust inference given our relatively small sample size (McElreath, 2020). Following recent suggestions for more informative reporting of statistical models (McShane et al., 2019), multiple estimates are provided to summarize the posterior distributions of model parameters. In particular, we report the median posterior effect size (i.e., *p* for median probabilities, *β* for median logistic regression coefficients, and LogOR for median log odds ratios), the median absolute deviation (MAD) as a robust measure of statistical uncertainty around median estimates, the 90% credible interval (CI), and the posterior probability of a positive effect (i.e., $pp_{+}$). When applicable, we also report Cohen's *d* to provide standardized effect sizes for comparison within and among studies. Note that, in contrast to classical *p*‐values indicating the probability of observing data given a null hypothesis $p(\mathrm{data}|H_{0})$, the reported $pp_{+}$ directly quantify the probability of a positive effect given the observed data $p(H_{+}|\mathrm{data})$. Values close to 1 thus indicate support for a positive effect, while values close to 0 indicate support for a negative effect. We interpreted effects with 90% CI excluding the null value (i.e., *p* = .5, *β* = 0, LogOR = 0) as providing clear support for a directional effect, which reflects a posterior probability of at least 0.95 in the expected direction (or alternatively ≤ 0.05 probability for an effect in the opposite direction).

Our sample size was determined by the availability of participants. Therefore, given the sample size, we estimated our statistical power for univariate comparisons of average grammar acquisition between deaf and hearing participants using a priori effect size criteria. To determine our a priori power for univariate comparisons of average grammar acquisition between deaf and hearing participants, we conducted a simulation‐based power analysis (Johnson et al., 2015). Specifically, we generated 200 random datasets assuming a small effect size for the mean difference between groups (Cohen's *d* = 0.3) on the transformed scale of our logistic regression model. Fifteen participant responses were simulated across 87 trials each for a single grammar, with modest between‐individual residual variance (*σ* ^2^ = 0.25) in mean probabilities. We then analyzed these datasets with Bayesian regression models, following the approach taken for our empirical analyses. Power was assessed by the proportion of simulated datasets for which the regression models were able to recover the simulated group difference with a posterior probability of >95% (or, in other words, where the 90% credible intervals for the slope estimate did not overlap with 0). Under these conditions, statistical power was desirably high (0.79). This suggests that our sample size and repeated measures design provided sufficient data to detect clear differences in performance between groups.

#### 3.3.1 Induction of the target grammar

**Fig. 5:**
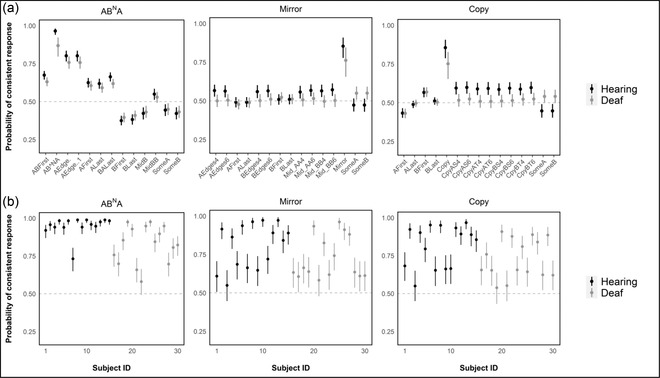
Average and individual‐level probabilities of grammar‐consistent responses. Posterior probabilities are summarized by the median estimate (dot) and 90% credible interval (lines) for hearing (black) and deaf (gray) participants. (a) The estimated probability of responding consistently with the target (AB^N^A, Mirror, Copy) and alternate grammars for an average hearing and deaf participant on a random trial. (b) Individual‐level probabilities of responding consistently with the target grammar on a random trial.

On average, both hearing and deaf participants performance was consistent with induction of the target grammars (Fig. 5a), as evidenced by their overall high probabilities of responding appropriately to the target grammars on any random trial for AB^N^A (hearing: *p* = .97 [MAD = 0.01], 90% CI [0.94, 0.98], $pp_{+}$= 1.00; deaf: *p* = .87 [0.04], 90% CI [0.80, 0.92], $pp_{+}$= 1.00), Mirror (hearing: *p* = .85 [0.04], 90% CI [0.78, 0.91], $pp_{+}$= 1.00; deaf: *p* = .76 [0.06], 90% CI [0.66, 0.84], $pp_{+}$= 0.99), and Copy grammars (hearing: *p* = .85 [0.03], 90% CI [0.79, 0.90], $pp_{+}$= 1.00; deaf: *p* = .75 [0.05], 90% CI [0.66, 0.83], $pp_{+}$= 0.99). Hearing participants were found to have a slightly higher probability of applying the target grammar on any random trial for the Copy grammar (*β* = 0.66 [0.38], 90% CI [0.02, 1.32], $pp_{+}$ = 0.95, Cohen's *d* = 0.37), although the effect size of this difference is notably small. A similarly small and statistically uncertain difference was estimated between hearing and deaf participants for the Mirror grammar (*β* = 0.60 [0.44], 90% CI [−0.12, 0.28], $pp_{+}$ = 0.91, *d* = 0.33). In contrast, there was a larger difference in average performance estimated for the AB^N^A target grammar (*β* = 1.44 [0.47], 90% CI [0.65, 2.22], $pp_{+}$ = 0.99, *d* = 0.79), such that hearing participants were more likely to correctly apply the AB^N^A grammar on average. Some alternate grammars also exhibited evidence of above‐chance consistency on average across both groups (Fig. 5a). Nonetheless, both hearing and deaf participants were more likely to respond consistently with the target AB^N^A (hearing: LogOR = 1.94–3.84, $pp_{+}$ ≥ 0.99; deaf: LogOR = 0.75–2.33, $pp_{+}$ ≥ 0.99), Mirror (hearing: LogOR = 1.47–1.87, $pp_{+}$ ≥ 0.99; deaf: LogOR = 0.96–1.26, $pp_{+}$ ≥ 0.99), and Copy grammars (hearing: LogOR = 1.38–2.04, $pp_{+}$ ≥ 0.99; deaf: LogOR range: 0.83–1.38, $pp_{+}$ ≥ 0.99), as compared to the alternate grammars.

Individual‐level random intercepts (Fig. 5b) further suggested that the majority of hearing and deaf participants exhibited greater than chance consistency (i.e., trial probability *p* > .5 with $pp_{+}$ ≥ 0.95) with the target grammar across all grammars: AB^N^A (hearing: 100% participants; deaf: 93%), Mirror (hearing: 93%; deaf: 93%), and Copy grammars (hearing: 93%; deaf: 87%). More importantly, the majority of hearing and deaf participants responded more consistently with the target grammar than any other alternate grammar (LogOR > 0 with $pp_{+}$ ≥ 0.95) across the AB^N^A (hearing: 100%; deaf: 93%), Mirror (hearing: 93%; deaf: 93%), and Copy grammars (hearing: 93%; deaf: 87%).

#### 3.3.2 Comparison of stimulus properties

Despite both hearing and deaf participants providing strong evidence for induction of the target grammar, the overall greater performance of hearing participants for the AB^N^A and Copy grammars suggested that deaf participants may have responded differently to specific stimulus properties, such as whether a stimulus required recognition or generalization from the training stimuli (*N* = 2 and 3 vs. *N* = 4 and 6) or whether the stimulus was similar or dissimilar to the rule used for generating training stimuli. We therefore further explored whether interactions were present on average between trial‐level stimulus properties (recognition vs. generalization, similarity vs. dissimilarity) and group (hearing or deaf status) for the AB^N^A and Copy grammars. Individual‐level random slopes were further estimated for both stimulus properties to account for heterogeneity among participants.

Hearing participants were found to perform slightly worse on trials with similar as compared to dissimilar stimuli for the Copy target grammar (*β* = −0.58 [0.22], 90% CI [−0.95, −0.22], $pp_{+}$ = 0.01, *d* = −0.32), but no clear effect was observed for the AB^N^A grammar (*β* = −0.43 [0.33], 90% CI [−0.98, 0.18], $pp_{+}$ = 0.11, *d* = 0.24). Stimuli similarity also did not have a clear effect on deaf participants for either AB^N^A (*β* = 0.15 [0.19], 90% CI [−0.17, 0.47], $pp_{+}$ = 0.79, *d* = 0.09) or Copy grammars (*β* = 0.15 [0.14], 90% CI [−0.09, 0.40], $pp_{+}$ = 0.85, *d* = 0.08). As a consequence, the small difference observed between hearing and deaf participants for the Copy grammar could be solely attributed to the dissimilar stimuli (recognition: *β* = 1.11 [0.40], 90% CI [0.43, 1.79], $pp_{+}$ = 0.99, *d* = 0.61; generalization: *β* = 0.81 [0.40], 90% CI [0.17, 1.47], $pp_{+}$ = 0.98, *d* = 0.45), where hearing participants performed particularly well, with no clear difference observed between the groups for similar stimuli (recognition: *β* = 0.53 [0.40], 90% CI [−0.13, 1.21], $pp_{+}$ = 0.91, *d* = 0.30; generalization: *β* = 0.22 [0.40], 90% CI [−0.43, 0.91], $pp_{+}$ = 0.72, *d* = 0.13). Hearing participants also exhibited a very small and moderately uncertain tendency to perform better with stimuli requiring recognition rather than generalization for the AB^N^A grammar (*β* = 0.53 [0.35], 90% CI [−0.04, 1.12], $pp_{+}$ = 0.94, *d* = 0.29), which was not observed among deaf participants (*β* = 0.06 [0.19], 90% CI [−0.24, 0.42], $pp_{+}$ = 0.62, *d* = 0.03). Hearing participants nonetheless exhibited higher average performance than deaf participants regardless of whether AB^N^A stimuli required recognition (similar: *β* = 1.55 [0.59], 90% CI [0.61, 2.55], $pp_{+}$ = 0.99, *d* = 0.86; dissimilar: *β* = 1.97 [0.56], 90% CI [1.02, 2.93], $pp_{+}$ = 0.99, *d* = 1.08) or generalization (similar: *β* = 1.02 [0.53], 90% CI [0.16, 1.93], $pp_{+}$ = 0.98, *d* = 0.56; dissimilar: *β* = 1.43 [0.49], 90% CI [0.64, 2.28], $pp_{+}$ = 0.99, *d* = 0.79). No clear effect of recognition was observed during Copy grammar trials for either hearing (*β* = 0.29 [0.22], 90% CI [‐0.06, 0.66], $pp_{+}$ = 0.91, *d* = 0.16) or deaf participants (*β* = −0.20 [0.14], 90% CI [−0.43, 0.04], $pp_{+}$ = 0.08, *d* = −0.11).

## 4 Discussion

We evaluated the performance of 15 deaf and 15 hearing adults in a visual artificial grammar learning task, assessing implicit learning of three different grammars ranging in computational complexity from a regular grammar to a mildly context‐sensitive grammar. Learning was assessed through participants’ ability to accept grammatical strings and to reject ungrammatical foils. Test stimuli included sequences of the same length as those presented in the exposure phase and sequences of a different length, thus testing for rule generalization. We assessed visual artificial grammar learning in deaf participants in order to provide new data relevant to the evaluation of the auditory scaffolding hypothesis, which states that the ability to represent sequential patterns requires developmental support by hearing, predicting that congenital deafness should hinder the development of sequential processing in deaf individuals (Conway et al., 2009). Specifically, we assessed sequencing abilities by focusing on a higher level of abstraction of sequential knowledge compared to the previous studies assessing the auditory scaffolding hypothesis. Our results show that, as a group, deaf participants learned all three grammars: they were able to accept novel grammatical sequences and reject ungrammatical foils. Bayesian analyses indicate that they did so by inducing each specific target grammar, not adopting alternative strategies.

Individual performance analysis indicated that almost all deaf participants (14/15 participants) performed above chance level with novel strings of length *N* = 2 and *N* = 3 (the length of sequences of the exposure phase), demonstrating a clear ability to recognize the given grammatical patterns without explicit teaching. Furthermore, half of the participants generalized the rule to a different length *N* = 4, showing that they could effectively generalize the learned rules. Although the vast majority of deaf participants could generalize to *N* = 6 in the regular grammar, this was not the case for the supra‐regular grammars, where performance was at chance level for almost all deaf participants. It is important to stress that this failure to generalize to *N* = 6 by deaf participants does not represent a general inability to generalize, since they did so with *N* = 4 generalization. Rather, this failure may indicate that *N* = 6 sequences were too long to be tracked by the deaf population for reasons that we discuss below. All in all, the ability shown by the present group of deaf participants to acquire grammars of varying complexity, up to the mildly context‐sensitive level on the extended Chomsky hierarchy, provides clear evidence against the auditory scaffolding hypothesis, especially considering that our deaf participants were all born deaf, and all but one never used a cochlear implant.

All deaf participants used LIS as their preferred means of communication at the time of testing, but only half of them were native signers, namely they acquired LIS from birth in their family by interacting with other deaf signers. The small sample size did not allow us to directly compare the group of native with that of nonnative signers. However, we saw no clear evidence that one group performed better than the other, as we had no evidence for specific participants performing worse than the others. To some extent, this is in line with results from Hall et al. (2018), which argued against a possible language scaffolding hypothesis for sequencing abilities. They found that implicit sequence learning capabilities develop both in deaf children with a delay in auditory and language exposure (deaf children of hearing parents with hearing restored thanks to cochlear implants), and in deaf children with no hearing exposure and no delay in language exposure (deaf children with a deaf parent). Nevertheless, further studies should focus on this aspect, taking into account not only age of exposure to language, but also each participant linguistic competence, an aspect that was not considered in the present group of participants and that we acknowledge as a limit of the present study.

**Fig. 6:**
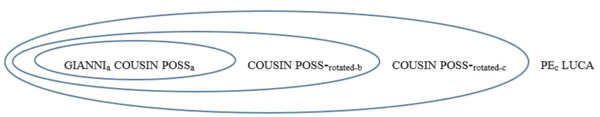
Set diagram of the sentence “the cousin of the cousin of the cousin of Gianni is Luca” in LIS (glosses, the video example is available on OSF: https://osf.io/25det/).

Another difficulty with the auditory scaffolding hypothesis is the presence of complex sequential patterns in sign languages. Even if it is true that sign languages rely more heavily on simultaneous information compared to spoken languages, this does not imply the absence of sequential processes in sign languages (Sandler, 1989), particularly in the syntactic domain. Because sign languages have been primarily developed by individuals with auditory deprivation, who by that hypothesis should have limitations in processing sequential sequences, the presence of such patterns cannot be easily explained. This is true also in the context of our experiment, as LIS, the language of our deaf participants, has been shown to contain complex syntactic dependencies. A sentence that exemplifies this is the LIS counterpart of a textbook example that illustrates the presence of recursive structure in natural languages, namely a sentence like the following: “gianni_a_ cousin poss_a_ cousin poss‐_rotated‐b_ cousin poss‐_rotated‐c_ pe_c_ luca” ^2^, which means ‘the cousin of the cousin of the cousin of Gianni is Luca’. We show the complex sequential structure of the subject noun phrase in this sentence by a set diagram (Fig. 6).

We also compared the performance of the deaf participants with that of a group of age‐ and education‐matched hearing participants to observe if the two groups used similar strategies to perform the task. Moreover, this allowed us to replicate the results obtained by Westphal‐Fitch et al. (2018) with a group of hearing adults with different characteristics. Westphal‐Fitch and colleagues’ participants were all university students, whereas the present group of hearing participants was a heterogeneous mix of people with different educational backgrounds, and most did not attend university. In essence, the results were replicated: this second group of participants also learned the three intended grammars and generalize the rules to lengths not encountered during the exposure phase, with only some difficulties in generalizing to *N* = 6 in supra‐regular grammars.

Regarding the deaf‐hearing comparison, both groups showed evidence of correctly identifying and applying the intended grammar, but the hearing group outperformed the deaf group in certain respects. This was especially true for the regular AB^N^A grammar and for the supra‐regular Copy grammar, and was driven by a difference in the willingness to reject ungrammatical strings, with hearing participants more likely to do so. Nonetheless, as discussed above, both groups mastered the grammars; therefore, this difference in performance should not be attributable to superior rule learning abilities in the hearing group compared to the deaf group, because deaf participants never performed worse on generalization relative to recognition. This confirms that deaf participants’ difficulties were not triggered by generalization sequences, which provide the most informative evidence for rule extraction.

As generalization sequences elicited a similar performance as recognition sequences, we hypothesize that the deaf‐hearing difference observed did not involve rule extraction, but rather some more general difficulty in encoding the incoming sequence in the deaf population. As previously pointed out by von Koss Torkildsen et al. (2018), verbal rehearsal strategies may have a relevant impact on sequence learning tasks performance. Sequence tracking may be more difficult for the deaf population due to visual stimulus interference with their verbal coding strategies. Hearing participants may have implemented some form of verbal (vocal) recoding to track the incoming sequence, for example, based on tile color (A tiles were gray/purple, whereas B tiles were red/green), or on the tiles’ internal shape (A tiles were composed of rounded shapes, B tiles by angular shapes). Such a recoding strategy for the AB^N^A grammar might run “round–angled–angled–angled–round.” Deaf participants attempting to implement such verbal encoding would suffer from interference, since verbal recoding of the experimental stimuli would need to use the same visual channel as their signed language. This hypothesis could be explored by future research, for example, comparing hearing and deaf participants using nonvisual (e.g., tactile) stimuli, or focusing on hearing participants and seeing if their performance will drop more if they perform the visual artificial grammar learning (AGL) task together with a verbal rehearsal task or with a task not tapping into verbal rehearsal. Another explanation that builds on verbal coding is that the sequence of recoded items would be a list of words for hearing participants and a list of signs for the hearing participants. As the sign span is known to be lower than the word span (including in LIS, cf. Geraci et al., 2008), deaf participants would be at a disadvantage, especially for longer lists.

In summary, the present work investigated visual artificial grammar learning abilities in deaf and hearing adults to test the auditory scaffolding hypothesis considering sequences with the same degree of abstraction as that required to process natural languages. We showed that both groups of participants could learn rules at different levels of the formal language hierarchy, thus providing new evidence against the auditory scaffolding hypothesis. A slight decrease in the performance of deaf participants compared to hearing participants was not attributable to differing rule extraction capabilities, but may result from interference during stimulus encoding.

## Supporting information

Supplementary information

## Notes

1 For the Mirror and the Copy grammar, N refers to the number of long‐distance dependencies, whereas for the ABNA grammar, *N* corresponds to the number of B elements. For example, AABBAA is a sequence with *N* = 3 generated by the Mirror grammar, AABABB is a sequence with *N* = 3 generated by the Copy grammar and ABBBA is a sequence with *N* = 3 generated by the ABNA grammar.

2 Following standard practice in the sign language literature, we indicate signs by small caps. Subscripts are used to indicate that two signs are articulated in the same position in the signing space; poss is a possessive pronoun, pe is a demonstrative pronoun and poss‐rotated is a special version of the poss sign that extends from the contralateral space to the ipsilateral space to spatially indicate the possession relation. See Mantovan (2020) for more information on possessive structure in LIS.

## References

[cogs13114-bib-0001] Arciuli, J. , & Simpson, I. C. (2011). Statistical learning in typically developing children: The role of age and speed of stimulus presentation. Developmental Science, 14(3), 464–473. 21477186 10.1111/j.1467-7687.2009.00937.x

[cogs13114-bib-0002] Arciuli, J. , & Simpson, I. C. (2012). Statistical learning is related to reading ability in children and adults. Cognitive Science, 36(2), 286–304. 21974775 10.1111/j.1551-6709.2011.01200.x

[cogs13114-bib-0003] Basso, A. , Capitani, E. , & Laiacona, M. (1987). Raven's coloured progressive matrices: Normative values on 305 adult normal controls. Functional Neurology, 2(2), 189–194. 3666548

[cogs13114-bib-0004] Bürkner, P. (2018). Advanced Bayesian Multilevel Modeling with the R Package brms. The R Journal, 10(1), 395–411. doi: 10.32614/RJ-2018-017

[cogs13114-bib-0005] Carpenter, B. , Gelman, A. , Hoffman, M. D. , Lee, D. , Goodrich, B. , Betancourt, M. , Brubaker, M. , Guo, J. , Li, P. , & Riddell, A. (2017). Stan: A probabilistic programming language. Journal of statistical software, 76(1), 1–32. 10.18637/jss.v076.i01 PMC9788645 36568334

[cogs13114-bib-0006] Conway, C. M. , Pisoni, D. B. , & Kronenberger, W. G. (2009). The importance of sound for cognitive sequencing abilities: The auditory scaffolding hypothesis. Current Directions in Psychological Science, 18(5), 275–279. 10.1111/j.1467-8721.2009.01651.x 20725604 PMC2923391

[cogs13114-bib-0007] Conway, C. M. , Pisoni, D. B. , Anaya, E. M. , Karpicke, J. , & Henning, S. C. (2011). Implicit sequence learning in deaf children with cochlear implants. Developmental Science, 14(1), 69–82. 10.1111/j.1467-7687.2010.00960.x 21159089 PMC3050521

[cogs13114-bib-0008] Corsi, P. M. , & Michael, P. (1972). Human memory and the medial temporal region of the brain (Vol. 34). McGill University Montreal.

[cogs13114-bib-0009] Dehaene, S. , Meyniel, F. , Wacongne, C. , Wang, L. , & Pallier, C. (2015). The neural representation of sequences: From transition probabilities to algebraic patterns and linguistic trees. Neuron, 88(1), 2–19. 26447569 10.1016/j.neuron.2015.09.019

[cogs13114-bib-0010] Fitch, W. T. , & Friederici, A. D. (2012). Artificial grammar learning meets formal language theory: An overview, Philosophical Transactions of The Royal Society B, 367(1598), 1933–1955. doi: 10.1098/rstb.2012.0103 PMC3367694 22688631

[cogs13114-bib-0011] Fitch, W. T. , & Hauser, M. D. (2004). Computational constraints on syntactic processing in a nonhuman primate. Science, 303(5656), 377–380. 10.1126/science.1089401 14726592

[cogs13114-bib-0012] Geraci, C. , Gozzi, M. , Papagno, C. , & Cecchetto, C. (2008). How grammar can cope with limited short‐term memory: Simultaneity and seriality in sign languages. Cognition, 106, 780–804. 10.1016/j.cognition.2007.04.014 17537417

[cogs13114-bib-0013] Giustolisi, B. , & Emmorey, K. (2018). Visual statistical learning with stimuli presented sequentially across space and time in deaf and hearing adults. Cognitive Science, 42(8), 3177–3190. 10.1111/cogs.12691 30320454 PMC6286205

[cogs13114-bib-0014] Gomez, R. L. (2002). Variability and detection of invariant structure. Psychological Science, 13(5), 431–436. 10.1111/1467-9280.00476 12219809

[cogs13114-bib-0015] Hall, M. L. , Eigsti, I. M. , Bortfeld, H. , & Lillo‐Martin, D. (2018). Auditory access, language access, and implicit sequence learning in deaf children. Developmental Science, 21(3), e12575. 10.1111/desc.12575 28557278 PMC8175005

[cogs13114-bib-0016] Jäger, G. , & Rogers, J. (2012). Formal language theory: Refining the Chomsky hierarchy. Philosophical Transactions of the Royal Society B: Biological Sciences, 367(1598), 1956–1970. 10.1098/rstb.2012.0077 PMC3367686 22688632

[cogs13114-bib-0017] Johnson, P. C. D. , Barry, S. E. , Ferguson, H. M. , & Müller, P. (2015). Power analysis for generalized linear mixed models in ecology and evolution. Methods in Ecology and Evolution, 6, 133–142. 10.1111/2041-210X.12306 25893088 PMC4394709

[cogs13114-bib-0018] Lashley, K. S. (1951). The problem of serial order in behavior. In L. Jeffress (Ed.), Cerebral mechanisms in behavior; the Hixon Symposium (pp. 112–136). Wiley.

[cogs13114-bib-0019] Mantovan, Lara (2020). Syntax: 14.2. Possessive phrases. In C. Branchini & L. Mantovan (Eds.), A grammar of Italian sign language (LIS) (1st ed.). Venezia: Fondazione Università Ca’ Foscari. 10.30687/978-88-6969-474-5

[cogs13114-bib-0020] Marcus, G. F. , Vijayan, S. , Rao, S. B. , & Vishton, P. M. (1999). Rule learning by seven‐month‐old infants. Science, 283(5398), 77–80. 10.1126/science.283.5398.77 9872745

[cogs13114-bib-0021] McElreath, R. (2020). Statistical rethinking: A Bayesian course with examples in R and Stan. CRC Press.

[cogs13114-bib-0022] McShane, B. B. , Gal, D. , Gelman, A. , Robert, C. , & Tackett, J. L. (2019). Abandon statistical significance. The American Statistician, 73(1), 235–245. 10.1080/00031305.2018.1527253

[cogs13114-bib-0023] Nissen, M. J. , & Bullemer, P. (1987). Attentional requirements of learning: Evidence from performance measures. Cognitive psychology, 19(1), 1–32.

[cogs13114-bib-0024] O'Donnell, T. J. , Hauser, M. D. , & Fitch, W. T. (2005). Using mathematical models of language experimentally. Trends in Cognitive Science, 9(6), 284–289. 10.1016/j.tics.2005.04.011 15925807

[cogs13114-bib-0025] R Core Team (2013). R: A language and environment for statistical computing. R foundation for statistical computing. http://www.R‐project.org/

[cogs13114-bib-0026] Raven, J. (1965). Guide to using the coloured matrices. Sets a, a b, b. HK Lewis.

[cogs13114-bib-0027] Reber, A. S. (1967). Implicit learning of artificial grammars. Journal of Verbal Learning and Verbal Behavior, 6(6), 855–863. 10.1016/S0022-5371(67)80149-X

[cogs13114-bib-0028] Saffran, J. R. , Aslin, R. N. , & Newport, E. L. (1996). Statistical learning by 8‐month‐old infants. Science, 274(5249), 1926–1928. 10.1126/science.274.5294.1926 8943209

[cogs13114-bib-0029] Saffran, J. , Pollak, S. D. , Seibel, R. , & Shkolnik, A. (2007). Dog is a dog is a dog: Infant rule learning is not specific to language. Cognition, 105(3), 669–680. 10.1016/j.cognition.2006.11.004 17188676 PMC2066190

[cogs13114-bib-0030] Saffran, J. R. (2002). Constraints on statistical language learning. Journal of Memory and Language, 47(1), 172–196. 10.1006/jmla.2001.2839

[cogs13114-bib-0031] Sandler, W. (1989). Phonological representation of the sign: Linearity and nonlinearity in American sign language. Foris.

[cogs13114-bib-0032] Shieber, S. M. (1985). Evidence against the context‐freeness of natural language. Linguistics and Philosophy, 8, 333–344. 10.1007/BF00630917

[cogs13114-bib-0033] Siegelman, N. , Bogaerts, L. , & Frost, R. (2017). Measuring individual differences in statistical learning: Current pitfalls and possible solutions. Behavior research methods, 49(2), 418–432. 26944577 10.3758/s13428-016-0719-z PMC5011036

[cogs13114-bib-0034] Stobbe, N. , Westphal‐Fitch, G. , Aust, U. , & Fitch, W. T. (2012). Visual artificial grammar learning: Comparative research on humans, kea (*Nestor notabilis*) and pigeons (*Columba livia*). Philosophical Transactions of the Royal Society B: Biological Sciences, 367(1598), 1995–2006. 10.1098/rstb.2012.0096 PMC3367688 22688635

[cogs13114-bib-0035] Terhune‐Cotter, B. P. , Conway, C. M. , & Dye, M. W. G. (2021). Visual sequence repetition learning is not impaired in signing DHH children, The Journal of Deaf Studies and Deaf Education, 26(3), 322–335. 10.1093/deafed/enab007 34017994

[cogs13114-bib-0036] Uddén, J. , Ingvar, M. , Hagoort, P. , & Petersson, K. M. (2012). Implicit acquisition of grammars with crossed and nested non‐adjacent dependencies: Investigating the push‐down stack model. Cognitive Science, 36(6), 1078–1101. 10.1111/j.1551-6709.2012.01235.x 22452530

[cogs13114-bib-0037] von Koss Torkildsen, J. , Arciuli, J. , Haukedal, C. L. , & Wie, O. B. (2018). Does a lack of auditory experience affect sequential learning? Cognition, 170, 123–129. 10.1016/j.cognition.2017.09.017 28988151

[cogs13114-bib-0038] Westphal‐Fitch, G. , Giustolisi, B. , Cecchetto, C. , Martin, J. S. , & Fitch, W. (2018). Artificial grammar learning capabilities in an abstract visual task match requirements for linguistic syntax. Frontiers in Psychology, 9, 1210. 10.3389/fpsyg.2018.01210 30087630 PMC6066649

