# Supplementary material for: Performance of Deaf Participants in an Abstract Visual Grammar Learning Task at Multiple Formal Levels: Evaluating the Auditory Scaffolding Hypothesis
Source: Cogn Sci. 2022 Feb 21;46(2):e13114. doi: 10.1111/cogs.13114 (PMC9286362; doi:10.1111/cogs.13114)
Supplement: Supplementary file 1 — Supplementary information [file COGS-46-0-s001.docx]

LIST OF ALTERNATIVE GRAMMARS

**Target grammar:**

AB^N^A

**Alternative grammars:**

ABFirst: any string starts with AB

AEdge+: any string with A on edges, with something in between

AEdge*: any string with A on edges, possibly nothing else

AFirst: any string that starts with A

ALast: any string that ends with A

BALast: any string that ends with BA

BFirst: any string that starts with B

BLast: any string that ends with B

Mid_B: any string that contains B, not at the edge

Mid_BB: any string that contains BB, not at the edge

SomeA: any string that contains A

SomeB: any string that contains B

**Target grammar:**

Mirror

**Alternative grammars:**

AEdges4: any string with N = 4 and with A on edges (A--A)

AEdges6: any string with N = 6 and with A on edges (A----A)

AFirst: any string that starts with A

ALast: any string that ends with A

BEdges4: any string with N = 4 and with B on edges (B--B)

BEdges6: any string with N = 6 and with B on edges (B----B)

BFirst: any string that starts with B

BLast: any string that ends with B

Mid_AA4: any string with N = 4 and with AA in 3^rd^ and 4^th^ positions (-AA-)

Mid_AA6: any string with N = 6 and with AA in 3^rd^ and 4^th^ positions (--AA--)

Mid_BB4: any string with N = 4 and with BB in 2^nd^ and 3^rd^ positions (-BB-)

Mid_BB6: any string with N = 6 and with BB in 3^rd^ and 4^th^ positions (--BB--)

SomeA: any string that contains A

SomeB: any string that contains B

**Target grammar:**

Copy

**Alternative grammars:**

AFirst: any string that starts with A

ALast: any string that ends with A

BFirst: any string that starts with B

BLast: any string that ends with B

CpyA^4: any string with N = 4 and with A in 1^st^ and 3^rd^ positions (A-A-)

CpyA^6: any string with N = 6 and with A in 1^st^ and 4^th^ positions (A--A--)

CpyA$4: any string with N = 4 and with A in 2^nd^ and 4^th^ positions (-A-A)

CpyA$6: any string with N = 6 and with A in 3^rd^ and 6^th^ positions (--A--A)

CpyB^4: any string with N = 4 and with B in 1^st^ and 3^rd^ positions (B-B-)

CpyB^6: any string with N = 6 and with B in 1^st^ and 4^th^ positions (B--B--)

CpyB$4: any string with N = 4 and with B in 2^nd^ and 4^th^ positions (-B-B)

CpyB$6: any string with N = 6 and with B in 3^rd^ and 6^th^ positions (--B--B)

SomeA: any string that contains A

SomeB: any string that contains B
